# New Appliances and Things Medical

**Published:** 1893-12-09

**Authors:** 


### NEW APPLIANCES AND THINGS MEDICAL.

[All preparations, appliances, novelties, &c., of which a notice is desired, should be sent for the Editor, to care of The Manager, 428, Strand, London, W.C.]

### HARVEST'S LENTIL FOOD.

The secrets of nourishing and inexpensive diets are getting better understood every day. Lentils are in the first rank in point of nourishing food stuffs, and few materials lend themselves more readily to a variety of uses. Few better soups can be produced for winter fare than those in which lentils play a large part. In the use of lentils plain and simple we are however somewhat in the hands of an indifferent cook. In the use of such preparations, such as Harvest's Lentil Food, much difficulty is obviated. The meal is beautifully smooth in use, and more easily cooked in all ways. Though it is above all suited for use in soups, it is also excellent in custards, puddings, and biscuits, and Messrs Harvest deserve the thanks of all housekeepers for an excellently-prepared article which is convenient, economical, and nourishing, a shilling tin of which will provide ten quarts of soup.

